# Supplementary material for: Flora Checklist in the Bayanaul State National Nature Park, Kazakhstan with Special Focus on New Species of Conservation Interest
Source: Plants (Basel). 2025 Apr 3;14(7):1119. doi: 10.3390/plants14071119 (PMC11991007; doi:10.3390/plants14071119)
Supplement: Supplementary file 1 [file plants-14-01119-s001.zip › plants-3524907-supplementary.pdf]

Supplementary Table S1: Detail floristic list of the Bayanaul State National Natural Park (BSNNP), Kazakhstan

s	Division	Class	Family	Species	Habitat
1	EQUISETOPHYTA	EQUISETOPSIDA	Equisetaceae Rich, ex DC	*Equisetum hyemale* L	in damp birch-pine and aspen-birch forests, less often in pine forests.
2				*Equisetum limosum* L.	in water, along the banks of mountain streams, in swampsin empty places.
3				*Equisetum palustre* L.	in damp aldersin pine, aspen and birch forests.
4				*Equisetum sylvaticum* L.	in wet axesnew, birch, pine, and more often alder forests.
5	POLYPODIOPHYTA	POLYPODIOPSIDA	Polypodiaceae Bercht. et C. Presl	*Polypodium vulgare* L.	granite in crevicesrocks, in pine forests on rocky slopes.
6			Thelypteridaceae Pichi Sermolli	*Thelypteris palustris* Schott *[Dryopteris the- lipteris* A. Gray]	in alder forests, along the banks of streams.
7			Aspleniaceae Mett, ex Frank	*Asplenium septentrionale* (L.) Hoffm.	in the racecracks in granite slabs.
8			Athyriaceae Alston	*Athyrium filix-femina* (L.) Roth	in the alder forestkah, along the banks of mountain streams.
9				*Cystopteris fragilis* (L.) Bernh. [C. *filix-fragilis* (L.) Bobr.]	in the cracks of granite rocks, under the stonesnyami, in sparse pine forests, alder forests.
10			Aspidiaceae Mett, ex Frank	*Dryopteris filix-mas* (L.) Schott	in shady places under blocks of granite slabs, in aspen and birch forests.
11				*Gymnocarpium robertianum* (Hoffm.) Newm.	in the crevices of granite slabs.
12				*Gymnocarpium tenuipes* Pojark. Ex Schmakov	in the cracks of granite slabs, in pine forests

13				*Gymnocarpium continentale* (V. Petrov) Pojark.	in the crevices of granite boulders.
14			Onocleaceae Pichi Sermolli	*Matteuccia struthiopteris* (L.) Tod. *[Struthiopt- eris filicastrum* All.]	in damp alder forests along the banks of mountain streams.
15			Woodsiaceae (Diels) Herter	*Woodsia ilvensis* (L.) R.Br.	in rock crevices, in sparse pine forests.
16	PINOPHYTA	PINOPSIDA	Pinaceae Lindl.	*Pinus sylvestris* L.	along the rocky slopes of the granite lowlands throughout the territory of the massifsiva.
17			Cupressaceae Bartl	*Juniperus sabina* L.	in cracks in granite blocks, on outcrops of granite rocks.
18		GNETOPSIDA	Ephedraceae Dumort.	*Ephedra distachya* L.	in cracks in granite rocks in sparse pine forests, on rocky slopes, in thickets of steppe shrubs.
19	MAGNOLIOPHYTA	MAGNOLIOPSIDA	Ranunculaceae Juss.	*Aconitum septentrionale* Koelle	along the edges of small-leaved forests.
20				*Adonis vernalis* L.	in the steppes, on steppe meadows, on the outskirts of forests and in shrubby areasgrowing.
21				*Adonis wolgensis* Steven	in the steppes along the slopewe have hills.
22				*Anemone sylvestris* L.	along forest edges, in damp pine forests, in meadows.
23				*Ceratocephala falcatus* (L.) Pers.	on emptyin the countryside, on degraded pastures, in the steppes, and thickets of bushes.

24	*Delphinium elatum* L.	in alder forests near water, on the outskirts of birch and aspen forests, in thickets of bushes.
25	*Halerpestes sasuginosa* (Pall.) Green.	along the shore of the lake Toraigyr.
26	*Pulsatilla patens* (L.) Mill.	on mountain slopes, in pine forests, on steppe meadows, in thepah.
27	*Ranunculus auricomus* L.	in damp meadows, on the edges of forests.
28	*Ranunculus lingua* L.	in alder and aspen-birch forests along the banks of rivers, in willow thickets.
29	*Ranunculus pedatus* Waldst. et Kit.	in olin the khovniki, near springs, in the valleys, on the steppemeadows.
30	*Ranunculus polyanthemos* L.	in alder and willow groves along the banks of rivers, sometimes in dampin forests and along roads.
31	*Ranunculus polyrhizos* Steph.	on the stoneon flat slopes, in thickets of bushes.
32	*Ranunculus sceleratus* L.	in alder and willow groves along the banks of rivers, sometimes in damp forests and along roads.
33	*Thalictrum flavum* L.	in the busheskov, in meadows, on the outskirts of aspen-birch forests.

34		*Thalictrum foetidum* L.	in the pine forests on the kamamuddy and stony slopes, in thickets of bushesnikov, in the steppes.
35		*Thalictrum collinum* Wallr.	in the bushes of kuold trees, in pine forests, in willow forests, in damp meadows.
36		*Thalictrum simplexL.*	in the busheskov, in damp birch, aspen and pine forestsin the forests, sometimes on the steppe slopes.
37	Berberidaceae Juss	*Berberis sibirica* Pall.	along rocky slopesto us hills, between stones and granite slabs, under cliffs.
38	Papaveraceae Juss.	*Chelidonium majus* L.	in shady places, in valleys, in alder forests, in bush thicketskov, in pine forests under the rocks.
39	Fumariaceae DC.	*Corydalis schanginii* (Pall.) B.Fedtsch.	on the outskirts of alder groves, near paths, under tall bushes.
40		*Fumaria officinalis* L.	granite along cracksslabs in pine forests and in thickets of steppe bushesold people
41		*Fumaria schleicheri* Sou.-Willem.	into small piecesearthy areas among granite rocks along the western shore of Lake Zhasybay.
42	Caryophyllaceae Juss	*Cerastium arvense* L.	on the slopes, in the pine forestin the pine and birch forests, in the steppes.

43	*Cerastium bungeanum* Vved. [C. *falcatum* Bunge]	in alder and willow forests, in river floodplains, in damp pine and birch forests, on grassy slopes.
44	*Dianthus campestris* M.Bieb.	in the steppes, innew pine forests, in shrub thickets.
45	*Dianthus leptopetalus* Willd.	along the edges of birch forests.
46	*Dianthus rigidus* M.Bieb.	in rock crevices, on dry slopes, in steppe meadows.
47	*Dianthus versicolor* Fisch, ex Link.	by the edgesmall-leaved forest stones.
48	*Arenaria koriniana* Fisch, ex Fensl	by steppeon the slopes, in the steppes.
49	*Arenaria longifolia M.* Bieb.	in steppe meadows, in thickets of bushes, in steppes.
50	*Arenaria saxatilis* L. [A. *stenophylla* Ledeb.]	on rocks, in sparse forests, in thickets of bushes.
51	*Gypsophila altissima* L.	in the steppes, on the steppein the meadows, in the thickets of bushes.
52	*Gypsophila muralis* L.	on the rockychvakh, on steppe meadows, sometimes on salt licksin cottony places.
53	*Gypsophila paniculata* L.	in the steppes, in the valleyson mountain rivers, on granite outcrops.
54	*Gypsophila patrinii* Ser.	in the steppes, on the Kamalow slopes, in bushes.
55	*Herniaria glabra* L.	on rocky slopes of low mountains, in cracks of granite slabs

56	*Melandrium album* (Mill.) Garcke	to the bushin pine forests and pine thickets.
57	*Melandrium viscosum* (L.) Celak.	in the steppes, on gravelly slopes, in sparse pine forests.
58	*Moehringia trinervia* (L.) Clairv.	in the alder forestke in the vicinity of Lake Zhasybay.
59	*Myosoton aquaticum* (L.) Moench *[Malachium aqaticum* (L.) Fries]	along the banks of rivers, in alder forestsin nikah, willow groves, and damp birch forests.
60	*Silene altaica* Pers. [5. *fruticulosa* (Pall.) Schis-chk.]	in cracks of granite slabs, on rubbleon the soft slopes.
61	*Silene baschkirorum* Janisch.	on steppe slopes.
62	*Silene chlorantha* (Willd.) Ehrh.	in the pine forests
63	*Silene graminifolia* Otth.	in the cracks of the stonethread slabs, under the stones.
64	*Silene media* (Litv.) Kleopow	in the steppes, on rocky slopes.
65	*Silene nutans* L.	in steppe meadows, inin new sparse forests.
66	*Silene repens* Patrin	in steppe meadows, along the banks of mountain rivers, in grassy pine forests.
67	*Silene venosa* (Gilib.) Aschers. [S. *latifolia* (Mill.) Rendle; *Oberna behen* (L.) Ikonn.]	on the moongah, in the valleys of streams, in alder forests.
68	*Silene wolgensis* (Hornem.) Besser ex Spreng.	in steppe meadows, in steppes, in shrubberyin the thickets.
69	*Stellaria bungeana* Fenzl	about the gun takeof the forest.

70		*Stellaria graminea* L.	in meadows, in thickets of bushes, in alder forests, in damp pine forestsin the forests.
71		*Stellaria media* (L.) Vill.	on about guns, on beRegam of rivers
72		*Stellaria palustris* Retz.	around the springs
73	Amaranthaceae Juss	*Amaranthus albus* L.	by the roads.
74		*Amaranthus retroflexus* L.	like weeds by the roads.
75	Chenopodiaceae Vent	*Artiplex laevis* C.A. Mey.	along the bankslazy lakes, often in weedy places.
76		*Atriplex patula* L.	in alder forests along the berethe hubbub of rivers, by the roads.
77		*Atriplex cana* C. A. Mey.	on salt marshes and solonetz.
78		*Atriplex tatarica* L.	near housing, in waste places.
79		*Atriplex verrucifera* Bieb.	on salt licks and withlonchaks
80		*Camphorosma monspeliaca* L.	on moderately and highly saline soils in steppe communitiessvakh.
81		*Ceratocarpus arenarius* L.	on the slopes of the mountainnitny hills.
82		*Chenopodium acuminatum* Willd.	on the rocklow soils in pine forests, along roads.
83		*Chenopodium album* L.	along roadsides, along forest edges.
84		*Chenopodium aristatum* L.	in the rocky steppes.
85		*Chenopodium glaucum* L	near housing, in waste places.
86		*Chenopodium hybridum* L.	on the stoneon flat slopes, in pine forests, in shrubby areasgrowing.
87		*Chenopodium rubrum* L.	on the sides of the roads

88		*Chenopodium urbicum* L.	through weedsstation, near the house.
89		*Kochia prostrata (L.) Schrad.*	on rocky and gravelly slopes of low mountains, on solonetzcottony soils.
90		*Kochia scoparia* (L.) Schrad.	near housing, in vacant lots, in wastelands.
91		*Krascheninnikovia ceratoides* (L.) Gueldenst. *[Eurothia ceratoides* (L.) C. A. Mey.]	by stepslopes.
92		*Petrosimonia triandra* (Pall.) Simonk.	according tolonchaks and salt licks.
93		*Polycnemum arvense* L.	on rocky slopes.
94		*Salsola collina* Pall.	weeds near housing
95	Polygonaceae Juss	*Atraphaxis frutescens* (L.) K.och	along the steppe slopes of the hills.
96		*Fagopyrum tataricum* (L.) Gaertn.	by threeamong the granite slabs.
97		*Polygonum alpinum* All. [Aconogon *alpinum* (All.) Schur]	meadow slopes, clearings, clearingspine forests.
98		*Polygonum amphibium* L.	It occurs in two forms: the aquatic form has a branched stemwhite up to 150 cm long and long-petiolate, floatingleaves on the surface of the water; the terrestrial form has a simple, erect stem, up to 70 cm high. It lives in stagnant and slow-moving bodies of water.
99		*Polygonum aviculare* L.	like weed at homehorn.

100	*Polygonum convolvulus* L.	in the bushes of kuold trees, in meadows, along river banks, in pine forestsin the crevices of granite slabs.
101	*Polygonum dumetorum* L.	along the valleys of ruwhose in alder forests, in thickets of bushes.
102	*Polygonum gracilis* (Ledeb.) Kick.	steppe communities, old marmot forests, saline soils.
103	*Polygonum hydropiper* L.	in swampy and highly humid alder forests, along river banks and in damp meadows.
104	*Polygonum lapathifolia* L.	in a highly humidified environmentin the alder forests, along the banks and valleys of rivers.
105	*Polygonum minus* Huds.	in alder forests, along the banks of rivers and lakes, in damp meadows.
106	*Polygonumpatulum* M.Bieb.	in mixed-grass feather-grass steppes, on saline meadows.
107	*Rheum nanum* Siewers.	on a rocky slope along the northern shore of the lake. Toraigyr.
108	*Rumex acetosa* L.	along the banks of streams in Olkhovniki.
109	*Rumex aquaticus* L.	along the banks of rivers, lakes, in meadows.
110	*Rumex confertus* Willd.	on the steppe-covered slopesgah, in damp pine forests.
111	*Rumex crispus* L.	meadows, near springs.

112		*Rumex marschallianus* Reichenb.	banks behindpond, lakes, other bodies of water.
113		*Rumex stenophyllus* Ledeb.	near springs, along the banks of lakes and rivers, sometimes on solonetz soilthese soils.
114		*Rumex thyrsiflorus* Fingerh.	in steppe meadows, in thickets of bushes.
115	Limoniaceae Lincz	*Goniolimon speciosum* (L.) Boiss.	on the rockmuddy and stony slopes, in the steppes.
116		*Limonium gmelinii* (Willd.) K.untze	to the steppein mixed grass meadows, in feather grass andlyn steppes.
117	Betulaceae S.F. Gray	*Alnus glutinosa* (L.) Gaertn.	in intermountain humid gorges, along the banks of mountain rivers and springs.
118		*Betula pendula* Roth	in pine forests, alder forestsin aspen and arborvitae forests, sometimes forming pure banksringing pegs.
119		*Betula pubescens* Ehrh.	in humidified areasstakh mixed with alder, in the valleys of mountain rivers and springs.
120	Hypericaceae Juss	*Hypericum perforatum* L.	on grassy slopes, in shrub thickets, on steppein the meadows
121	Pyrolaceae Dumort	*Moneses uniflora* (L.) A.Gray	in the pine forests
122		*Orthilia secunda* (L.) House *[Ramischia secun- da* (L.) Garcke]	in pine-birch, aspenin the forests, in the alder groves

123		*Pyrola chlorantha* Sw.	in a pine forest on the bank of a drying up stream.
124		*Pyrola rotundifoliaL.*	in pine-birch, aspen forests, in alder forests.
125	Primulaceae Vent	*Androsace maxima* L. [A. *turczaninovii*Freyn.]	on rocky and gravelly slopes
126		*Androsace septentrionalis* L.	in the steppes, in thickets of bushes, on rocky slopesnah.
127		*Glaux maritima* L.	in saline meadows in iris thickets.
128		*Lysimachia vulgaris* L.	in alder forests, willowsin the birch and aspen forests along the banks of rivers
129		*Primula longiscapa* Ledeb.	in the meadows, along theregam of lakes, in thickets of iris.
130	Violaceae Batsch	*Viola canina* L.	on mountain slopes in pine forests, in shrub thickets.
131		*Viola collina* Bess.	in the sparse pine forestkah
132		*Viola montana* L. [V. *elatior* Fries.]	in pinewith shrub layer
133		*Viola hirta* L.	in alder forests, in pine forestssah, in bush thickets.
134		*Viola persicifolia* Schreb.	on wet soilin the meadows, in alder forests along the banks of rivers, in the cracks of rocks.
135		*Viola rupestris* F.W.Schmidt	on rocky slopes, under cliffs, in pine forests, in steppe meadows.
136	Tamaricaceae Link	*Tamarix ramosissima* Ledeb.	along the bankslazy lakes, often used in landscaping

137	Salicaceae Mirb	*Populus balsamifera* L.	widely usedis found in plantings around holiday homes and tourist centersical bases.
138		*Populus tremula* L.	in birch groves, in shady pine forests, in valleys.
139		*Salix alba* L.	in the valleys of mountain rivers
140		*Salix bebbiana* Sarg. (S. *starkeana* Willd.)	shore of the lake Zhasybay, sanatorium "Beryozka".
141		*Salix caprea* L.	forms thickets in the floodplains of streams.
142		*Salis caspica* Pall.	around the springs.
143		*Salix cinerea* L.	in river valleys among alders, around springs, in valleys between hills.
144		*Salix lapponum* L.	in the alder forests
145		*Salix pentandra* L.	in the valleys of streams, in theflood meadows.
146		*Salix rosmarinifolia* L.	on raw meadows, inlines of mountain rivers, mixed with alder.
147		*Salix triandra* L.	found along the banks of rivers and springs.
148	Brassicaceae Burnett	*Alyssum desertorum* Stapf	in the cracks of the stonenitov, on the steppe slopes, near the dwellings
149		*Alyssum lenense* Adams	on rocky slopesnah, in the steppes, in the thickets of steppe bushes
150		*Alyssum tortuosum* Waldst. et Kit. ex Willd.	in the steppes, on rocky slopes.

151	*Arabidopsis thaliana* (L.) Heynh.	in thickets of steppe shrubs, on rocky slopes, in the steppes.
152	*Arabiodopsis toxophylla* (M. Bieb.) N. Busch	around the springs.
153	*Arabis borealis* Andrz.	in alder forests, in damp pine forests, in bush thicketskov
154	*Arabis pendula* L.	in alder forests near water, in weedy places.
155	*Barbarea stricta* Andrz.	in damp meadows, in humid forests.
156	*Berteroa incana* (L.) DC.	in steppe meadows, in steppes, on rocky slopes, in pine forestsin the bushes and thickets.
157	*Bunias orientalis* L.	on fallow land, weedy
158	*Camelina microcarpa* Andrz.	on steppemeadows, in weedy places, along roads, onon the rolling mountain slopes.
159	*Capsella bursa-pastoris* (L.) Medikus	forlittered areas.
160	*Cardamine impatiens* L.	in alder forests, along the banks of rivers and streams.
161	*Cardamine parviflora* L.	in the valleys of streams
162	*Chorispora sibirica* (L.) DC.	on gravelly slopes, steppe meadows, on coastal areasin the ravines, by the roads.
163	*Chorispora tenella* (Pall.) DC.	on fallow lands, roadsides, in forest clearings.
164	*Clausia aprica* (Stephan) Korn.-Trotzky	in the steppes, on rocky and gravelly slopes, along roads and in steppe meadows.

165	*Descurainia sophia* (L.) Webb ex Prantl.	between granite slabs, near housing, in weedy places.
166	*Draba lanceolata* Royle [D. *cana* Rydb.]	gravelly and rocky slopes.
167	*Draba nemorosa* L.	along shady cracks in rocks, on rocky slopes, on steppe meadows.
168	*Erysimum cheiranthoides* L.	in dry meadows, along roads, on forest edges.
169	*Erysimum diffusum* Echrh. [E. *canescens* Roth]	steppe slopes
170	*Erysimum leucanthemum* (Steph.) B. Fedtsch.	steppe slopes
171	*Erysimum marschallianum* Andrz.	meadow steppes, steppe slopes.
172	*Erysimum sisymbrioides* C.A. Me y.	by beRegu lake Toraigyr.
173	*Isatis costata* C.A. Mey.	on rocky slopes, in rock crevices, along the shores of lakes
174	*Lepidium densiflorum* Schrad	near housing, in waste places.
175	*Lepidium latifolium* L.	around the springs, inof the fields, on slightly saline soil.
176	*Lepidium perfoliatum* L.	in saline steppes, in thickets of bushes, in steppe meadows.
177	*Lepidium ruderale* L.	in saline soilspah, along the shores of lakes, in littered areas.
178	*Neslia paniculata* (L.) Desv.	near housing, along granite cracks, along garbage areas.
179	*Rorippa palustris* (L.) Besser	in littered areas, along the banks of lakes and rivers.

180		*Sisymbrium loselii Jusl.*	near housing, in waste places.
181		*Sisymbrium polymorphum* (Murray) Roth	in thickets of steppe shrubs, on gravelly and rocky slopes, in dry pine forests.
182		*Thlaspi arvense* L.	in the thickets of steppe bushesold trees, on slopes, in steppe meadows.
183		*Turritis glabra* L.	in steppe meadows, in thickets of steppe shrubs, in rock crevices, in pine forests.
184	Malvaceae Juss	*Althaea officinalis* L.	natural meadows.
185		*Lavatera thuringiaca* L.	on the outskirts of Olgroves and birch forests, in meadows, in bushesold people.
186		*Malva pusilla* Sm.	in littered areas, near roads.
187	Ulmaceae Mirb.	*Ulmuspumila* L.	a common tree in plantings, along roadsides
188	Cannabaceae Endl	*Cannabis ruderalis* Janisch.	along the edge of the aldersniks, in pine forests, along roads.
189		*Humulus lupulus* L.	in alders, in uramah, along the valleys of mountain rivers.
190	Urticaceae Juss	*Urtica dioica* L.	in alder forests, thickets of bushes, along roads.
191		*Urtica urens* L.	in vegetable gardens, near houses
192	Euphorbiaceae Juss	*Euphorbia gmelinii* Steud.	in the cracks of the stonethread slabs in the western part of the park.
193		*Euphorbia humilis* C.A. Mey.	in pine forests, in cracks in granite slabs.

194		*Euphorbia microcarpa* Prokh.	on the stoneflat slopes, along granite outcrops in the vicinity of the city of Au-It pours and the lake Zhasybay.
195		*Euphorbia subcordata* C.A. Mey. ex Ledeb.	on rocky soils.
196		*Euphorbia virgata* Waldst. et Kit.	naturegrass meadows, fallow lands, forest belt edges.
197	Crassulaceae DC.	*Orostachys spinosa* (L.) C.A. Mey.	on kain variegated soils in crevices of granite slabs, in dry pine forests.
198		*Sedum hybridum* L.	in the crevices of granite slabs, in pine forests, in shrub thickets.
199		*Sedum telephium* L.	in meadows, in alder forests along the banks of rivers, sometimes in pine forests.
200	Grossulariaceae DC.	*Grossularia acicularis* (Smith.) Spach.	35 km west of the village. Toraigyr, on the dump.
201		*Ribes aureum* Pursch	widely used in forest plantations, near housing.
202		*Ribes hispidulum* (Janez.) Pojark	in Olkhovnikah along the banks of streams, in shady gorges, sometimes in pine forests.
203		*Ribes nigrum* L.	in alder forests, at the bottoms of deep gorges, in damp pine forests.
204		*Ribes saxatile* Pall.	around lakes on granite boulders, on rocky slopes, in pine forests.

205	Parnassiaceae S. F. Gray	*Parnassia palustris* L.	across the meadows, around the familyniks, on slightly saline soils.
206	Rosaceae Juss	*Agrimonia pilosa* Ledeb.	in alder and birch forests.
207		*Cerasus tomentosa* (Thunb.) WaII.	on the rockflat slope in the vicinity lake Zhasybay.
208		*Chamaerhodos erecta* (L.) Bunge	on the rocklowland soils, in steppes and pine forests.
209		*Cotoneaster melanocarpus* Fisch. ex Blytt	on rocky slopes, in pine forests, ingrowths of bushes.
210		*Cotoneaster multiflora* Bunge	in the cracks of granite slabs on the slopes of Mount Auliet.
211		*Crataegus altaica* Ledeb. ex Loud	by daysdeep gorges, in alder forests, along the valleymountain rivers for us.
212		*Crataegus chlorocarpa* Lenne et K.Koch	in the cracks of granite slabs on the slopes of Mount Aupours
213		*Crataegus sanguinea* Pall.	in the floodplains of streams among shrub thickets.
214		*Filipendula ulmaria* (L.) Maxim.	in the alder forestin the swales and willows along the banks of streams, in humidin special places.
215		*Filipendula vulgaris* Moench [F. *hexapetala* Gilib.]	in the steppes, on steppe meadows, in the bushin old growth thickets, along the edges of forests.
216		*Fragaria vesca* L.	in birch-aspen and shady pine forests, in alder forests.

217	*Fragaria viridis* (Duchesne) Weston	in steppe meadows, in shrub thickets, on the edges of pine forests.
218	*Geum aleppicum* Jacq.	in the meadows, on the edgeKamkolkov, at the springs [Karamysheva, Rachkov-[Skaia, 1973].
219	*Geum urbanum* L.	in alder forests along the berethe din of mountain rivers, in bush thickets, along roads.
220	*Padus* avium Mill.	in the valleys of rivers and streams, in alder forests.
221	*PotentillaacaulisL.*	in caragana-oat and wormwood steppes, on rocky peaks withpok.
222	*Potentilla anserina* L.	in wet meadows, along the shores of lakes.
223	*Potentilla argentea* L.	in the steppes, in pine forests, in bush thickets, in granite crevicesslabs
224	*Potentilla bifurca* L.	in the steppes, on the steppein meadows, in thickets of bushes.
225	*Potentilla fruticosa* L. *[Dasiphora fruticosa* (L.) Rydb.]	in the cracks of granite slabs, on the stoneon the soft slopes.
226	*Potentilla humifusa* Willd. ex Schltdl.	in steppe meadows, in feather grass steppes, inin bushes and pine forests.
227	*Potentilla impolita* Wahlenb.	on grassy slopes of low mountains, in steppe meadows, in steppes.

228	*Potentilla parvifolia* Fisch, ex Lehm. [Dasy- *phora parvifolia* (Fisch.) Juz.] -	in the cracks of the stonethread slabs on rocky slopes.
229	*Potentilla transcaspia* Th. Wolf.	on the stoneon stony and gravelly soils, in steppes, in thickets of bushes.
230	*Potentilla supina* L.	in the meadows.
231	*Potentilla virgata* Lehm. [P. *dealbata* Bunge]	along the shore of the lake Torayagyr.
232	*Rosa acicularis* Lindl.	in pine forests, on forest edges, in shrub thickets, on slopes, in alder forests.
233	*Rosa glabrifolia* C.A.Mey. ex Rupr.	in steppe meadows, in shrub thickets, in alder forests, along the banks of rivers and springs.
234	*Rosa laxa* Refz	in alder forests, along the banks of rivers, in pine forests.
235	*Rosa majalis* Herrm.	in pine forests, on steppe meadows.
236	*Rosa spinosissima* L.	in the bushespyah, in pine forests.
237	*Rubus caesius* L.	in the floodplains of streams
238	*Rubus idaeus* L.	in alder forests along the banks of rivers, on slopes, in pine forests, in crevices of granite slabs.
239	*Rubus saxatilis* L.	in birch and pine forests, on rocky slopes.
240	*Sanguisorba officinalis* L.	in the meadows, grassyon flat slopes, in shrubby steppes.

241		*Sorbus sibirica* Hedl.	in pine forests with undergrowth.
242		*Spiraea crenata* L.	in the steppes, among the bushesnikov, on rocky scree.
243		*Spiraea hypericifolia* L.	in shrub thickets, in steppes, on rocky slopes, in pine forests.
244	*Lythraceae J. St.-Hil*	*Lythrum salicaria* L.	along the banks of streams in alder forests.
245		*Lythrum virgatum* L.	in alder and willow grovesalong the banks of rivers and lakes.
246	Onagraceae Juss	*Chamaenerion angustifolium* (L.) Scop.	on kaon sandy and gravelly slopes, in pine forests, inin bushes, in cracks in granite slabs.
247		*Circaea alpina* L.	in alder forests at the bases of tree trunks, on deadwood and rotting stumps.
248		*Circaea lutetiana* L.	in the valleys of streams in the blackalder groves.
249		*Epilobium hirsutum* L.	in alder forests, in willowsin the ravines near rivers and streams.
250		*Epilobium palustre* L.	in alder and willow grovesnear the water, along the shores of lakes.
251		*Epilobium roseum* Schreb.	floodplains of streams, around springs.
252		*Epilobium tetragonum* L. [E. *adnatum* Griseb.]	in alder and willow groves, in damp gorgeslyakh.
253	Fabaceae Lindl	*Astragalus alopecurus* Pall.	along the roadsides, on fallow lands.

254	*Astragalus buchtormensis* Pall.	on the stoneon a hundred slopes along the northern shore of Lake Toray-gyr
255	*Astragalus cornutus* Pall.	along the steppe slopes of the hills.
256	*Astragalus danicus* Refz.	natural meadows
257	*Astragalus kasachstanicus* Golosk.	crushed stonesflat slopes.
258	*Astragalus onobrychis* L.	meadow steppes on the slopes of hills.
259	*Astragalus penduliflorus* Lam. [A. *propinquus Schischk.]*	in the meadows
260	*Astragalus sulcatus* L.	in meadows, on slopes.
261	*Astragalus testiculatus* Pall.	in the steppes, in thein the growths of steppe shrubs, in steppe meadows.
262	*Astragalus vulpinus* Willd.	natural meadows, edges of small-leaved forests.
263	*Caragana arborescens* Lam.	widely usedused in landscaping.
264	*Caragana frutex* (L.) C. Koch	bushthickets on the steppe slopes of granite lowlands.
265	*Glycyrrhiza korshinskyi* Grig.	in the meadows and steppes.
266	*Glycyrrhiza uralensis* Fisch.	on the solonetzin the meadows, on the edges of pine forests, in the steppes, along the roads.
267	*Hedysarum gmelinii* Ledeb.	along the rocky and gravelly slopes of the hills.
268	*Lathyrus palustris* L.	in the alder forests, in the willowsin damp pine forests.

269	*Lathyrus pisiformis* L.	in meadows, in thickets of bushes, in pine forests.
270	*Lathyrus pratensis* L.	in the alder forests, on the moongah, in the bushes.
271	*Lathyrus tuberosus* L.	in the steppes, on steppe meadows, in thickets of bushes.
272	*Lotus praetermissus* Kupr.	in thickets of steppe shrubs, in damp meadows.
273	*Lotus sergievskiae* R. Kam. et Kovalevsk. *(Lotus frondosus* (Freyn) Kuprian.)	in meadows, along the banks of rivers and lakes.
274	*Medicago falcata* L.	in steppe meadows, in thickets of bushes in pine forests.
275	*Medicago lupulina* L.	in meadows, in thickets of bushes, along roads, along the banks of rivers.
276	*Medicago romanica* Prodrn	in pine forests, on steppe meadows, in thickets of bushes.
277	*Melilotus albus* Medikus	on grassy slopes, in meadows.
278	*Melilotus dentatus* (Waldst. et Kit.) Pers.	in meadows, along the banks of lakes and rivers.
279	*Melilotus officinalis* (L.) Pall.	near roads.
280	*Onobrychis arenaria* (Kit.) DC. [O. *tanaitica* Spreng.]	on gravelly slopes, steppe meadows, in shrub thickets, on forest edges.
281	*Oxytropis brevicaulis* Ledeb.	on steppemeadows, on rocky and gravelly slopes, in rock crevices.
282	*Oxytropis floribunda* (Pall.) DC.	on rocky and gravelly slopes, on steppe meadows.

283			*Oxytropis glabra* (Lam.) DC.	in steppe meadows, on the banks of rivers and lakes.
284			*Oxytropis pilosa* (L.) DC.	in the steppes, on steppe meadows, on slopes, in pine forests.
285			*Thermopsis lanceolata* R. Br.	along the road from the village to Lake Zhasybay.
286			*Trifolium lupinaster* L.	in pine forests, in birch forests, in alder forests, on steppe meadowsgah, in the bushes.
287			*Trifolium pratense* L.	natural meadows.
288			*Trifolium repens* L.	natural meadows
289			*Vicia cracca* L.	in alder forests, pine forests, willow forests, meadows, and shrub thickets.
290			*Vicia sepium* L.	on the slopes, in pine forests, in willowsin forests, in shrub thickets, in alder groves.
291			*Vicia tenuifolia* Roth	in steppe meadows, in thickets of bushes, in mixed-grass bedspah.
292		Aceraceae Juss.	*Acer negundo* L.	It is widely used in landscaping of populated areas, holiday homesha, tourist bases.
293		Linaceae S. F. Gray	*Linum pallescens Bunge*	along the roadsides tohorn, slopes of hills.
294		Geraniaceae Juss.	*Geranium collinum* Stephan ex Willd.	on the moongah, in the alder groves.
295			*Geranium divaricatum* Ehrh.	on rocky slopes, in thickets of bushes on the steppemeadows.

296		*Geranium pratense* L.	in alder forests, on the edges of birch-aspen forests, in meadows.
297		*Geranium pseudosibiricum* J.Mayer	in meadows, in mountain river valleys, in birch-pine forests.
298		*Geranium schrenkianum* Trautv. ex Pavlov	in alder forests, along the banks of rivers and springs, in damp meadows.
299		*Geranium sibiricum* L.	among the stones along theregam of lakes, in alder forests near the stream.
300	Balsaminaceae A. Rich.	*Impatiens noli-tangere* L.	in the shady olin the villages along the banks of mountain rivers.
301	Polygalaceae R. Вг.	*Polygala hybrida* DC.	in mixed-grass steppes, in pine forests, on the Kamelow mountain slopes.
302	Santhalaceae R. Вг.	*Thesium arvense* Horv.	on the steppe-covered slopesgah, along the rocky slopes.
303		*Thesium refractum* C.A. Mey.	in mixed-grass steppes, on rocky slopes.
304	Rhamnaceae	*Rhamnus cathartica* L.	in floodplains of streams, in thickets of bushes.
305	Elaeagnaceae Juss	*Elaeagnus oxycarpa* Schlecht.	widely distributedcommon in artificial plantings.
306	Apiaceae Lindl.	*Aegopodium podagraria* L.	in pine forests.
307		*Angelica palustris* (Besser) Hoffm. *[Ostericum palustre* Bess.]	in the black alder forest [Karamysheva, Rachkovskaya, 1973

308	*Carum carvi* L.	in damp grassy clearings, in alder forests along streams, in shrub thickets, in meadows.
309	*Cenolophium denudatum* (Hornem.) Tutin [Cfischeri (Spreng.) Koch, ex DC.]	on steppein meadows, in alder and willow groves, along the banks of rivers.
310	*Chaerophyllum prescottii* DC.	in the steppes, on steppe meadows, in thickets of bushes.
311	*Cicuta virosa* L.	in the alder forests along the riverwhose, sometimes in water, in marshy meadows.
312	*Cnidium dubium* (Schkuhr) Thell. *[Cadenia du- bia* (Schkuhr) Lavrova et V.N. Tikhon.]	to the steppein meadows, birch forests, and damp grassy pine forests.
313	*Conium maculatum* L.	wastelands, on the site of old sheepfolds, near housing.
314	*Eryngium planum* L.	in the steppes, along roads, in steppe meadows.
315	*Ferula soongarica* Pall, ex Spreng.	to the steppein the meadows, along the valleys between the hills, on the grasson shady slopes, on gravelly-fine earth trails.
316	*Ferula tatarica* Fisch.	in the steppes of the western partsti park, near the mountain "Baba Yaga".
317	*Heracleum sibiricum* L.	in alder forests along the banks of rivers, in willow thickets, in the bottoms of deep gorges.

318		*Oenanthe aquatica* (L.) Poir.	banks of waterlanguage, floodplains of streams.
319		*Peucedanum morisonii* Besser ex Spreng.	in the steppes, on the edges of pine forests, on the steppein the meadows, in the thickets of bushes.
320		*Seseli buchtormense* (Fisch. ex Hornem.) W.D.J.Koch *[Libanotis buchtormensis* (Fisch.) DC.]	on rocky and gravelly slopes innew forests, in the cracks of granite slabs.
321		*Seseli krylovii* (V. Tichomirov) M. Pimen. et Sdobnina *[Libanotis sibirica* (L.) C.A. Mey.]	on the slopes in pine forests, in shrubby areasgrowing.
322		*Seseli ledebourii G.* Don	on the rockychvakh in feather-grass steppes, on steppe meadows.
323		*Seseli strictum* Ledeb.	meadows around relativescove.
324		*Silaum silaus* (L.) Schinz et Thell.	meadow steppes, clearings around springs.
325		*Sium sisaroideum* DC.	in aldersrivers, in deep gorges, in damp birch forests.
326		*Sium medium* Fisch, et Mey.	in alder and birch forests.
327	Caprifoliaceae Juss.	*Lonicera microphylla* Willd. ex Schult.	no rocky and rocky slopes in the crevicenah granite slabs.
328		*Lonicera pallasii* Ledeb.	on the edge of a birch and aspen forest, along the bank of a stream (L.V. Denisova) [Karamysheva, Rachkovskaya, 1973].

329			*Lonicera tatarica* L.	along the bottoms of deep gorges, in the steppes, in bush thickets.
330		Viburnaceae Rafin	*Viburnum opulus* L.	deep along the bottomsgorges, in the undergrowth of damp pine-birch forests, in alder forests along the banks of streams.
331		Valerianaceae Batsch	Patrinia intermedia (Hornem.) Roem. et Schult.	in pine forests, in crevices of granite slabs, on slopes, in the steppes.
332			*Valeriana tuberosa* L.	in the steppes, in thickets of steppe bushes, in ravines.
333		Dipsacaceae Juss.	*Scabiosa isetensis* L.	in the steppes, on rubbleon the soft slopes.
334			*Scabiosa ochroleuca* L.	in steppe meadows, in thickets of bushes, sometimes in pine forests.
335		Rubiaceae Juss.	*Galium aparine* L.	in thickets of bushes, in steppe meadows.
336			*Galium boreale* L.	in alder and willow groves along the banks of rivers and springs, in dampnew forests.
337			*Galium palustre* L.	natural meadows, stream floodplains.
338			*Galium verum* L.	in steppe meadows, in steppe shrub thickets, in pine forests on rocky slopes.
339			*Galium uliginosum* L.	through swamps and marshy banks of streams in deep gorges.

340	Gentianaceae Juss	*Anagallidium dichotomum* (L.) Griseb.	under rocks, in thickets of bushes, in sparsein the burnt-out pine forests.
341		*Gentiana barbata* Froel.	in the meadows, in the thicketsa patch of bushes at the foot of the slopes [of KaramaSheva, Rachkovskaya, 1973].
342		*Gentiana cruciata* L.	in meadows, in pine forests, in thickets of bushes.
343		*Gentiana decumbens* L.	in wet meadows [Karamysheva, Rachkovskaya, 1973].
344		*Gentiana fetisowii* Regel et C.Winkl.	on the moongah, on the slopes.
345		*Gentiana pneumonanthe* L.	meadows, around springs.
346		*Gentiana riparia* Kar. et Kir.	in damp, sometimes saline meadows [Karamysheva, Rach[Kovskaya, 1973].
347	Asclepiadaceae R. Вr.	*Vincetoxicum sibiricum* (L.) Decne.	on steppe and rocky slopes, in pine forests on rocky and gravelly soils.
348	Solanaceae Juss	*Solanum dulcamara* L.	in aldersregam of streams and springs, in thickets of bushescove.
349		*Solanum kitagawae* Schonb.-Tem. [S. *depila-tum* Kitagawa]	along the banks of the rivers in Olkhovnykah.
350		*Solanum nigrum* L.	in the alder forests, in the thicketsbushes, in weedy places.
351	Convolvulaceae Juss	*Calystegia sepium* (L.) R.Br.	in alder forests along the banks of mountain rivers, in willow groves, in thickets of bushes.

352		*Convolvulus arvensis* L.	in the steppes on the stonein soft soils, in pine forests, along roads.
353	Cuscutaceae Dumort	*Cuscuta approximata* Bab.	in alder forests, in pine forests, in shrub thickets.
354		*Cuscuta europaea* L.	in alder forests and withsnapshots.
355	Boraginaceae Juss.	*Arnebia decumbens* (Vent.) Coss. et Kralik	on rocky and gravelly slopes, in petrofit steppes.
356		*Asperugo procumlens* L.	through abandoned sheepfolds, old cattle camps, through overgrown areasmillion bushes.
357		*Cynoglossum officinale* L.	in alder forests, in willow thickets, sometimes along roads.
358		*Echium vulgare* L.	in littered areas, along roads, in wastelands.
359		*Eritrichium rupestre* (Pall.) Bunge	on crushed stoneon flat slopes, on steppe meadows.
360		*Hackelia deflexa* (Wahlenb.) Opiz	by the shadowon the flat slopes and gorges, in the alder forests, in thegrowths of bushes.
361		*Lappula myosotis* Moench [L. *echinata* Gilib.]	in alder forests along the banks of rivers, on litteredin special places.
362		*Lithospermum officinale* L.	in alder forests, in aspen forests, in shrub thickets.
363		*Miosotis caespitosa* K. F. Schultz	in floodplains of streams, in natural meadows.
364		*Myosotis micrantha* Pall, ex Lehm.	on the rockflat slopes in the surrounding lake Zhasybay.

365		*Myosotis palustris* (L.) L.	in alder forests near streams, in damp meadows, in willow thickets, along the shores of lakes
366		*Myosotis sparsiflora* Pohl	in the meadows, on the rocklow slopes, in pine forests.
367		*Nonea pulla* DC.	in the steppes, by the roads, in the weedsrented places.
368		*Onosma arenariumWMsl.* et Kit.	on the stoneon a hundred slopes along the northern shore of Lake Toraigyr.
369		*Onosma simplicissimum* L.	in the steppes, in dry pine forests on the slopes of low mountains, in shrubsin the thickets.
370		*Tournifortia sibirica* L. *[Argusia sibirica* (L.) Dendy, *Messerschmidia sibirica* (L.) L. fil.]	no roadsides, wastelands, damp places.
371	Scrophulariaceae Juss	*Dodartia orientalis* L.	on rocky and gravelly groundlow slopes, in the steppes, in the thickets of bushescove.
372		*Euphrasia pectinata* Ten. [*E. tatarica* Fisch. ex Spreng.]	in pine forests, in bush thickets, in weedy places
373		*Linaria acutiloba* Fischer	in the steppes, on dry landlow meadows, sparse pine forests, in threegranite slabs.
374		*Linaria vulgaris* Mill.	on rocky slopesNah, in the steppes, in the valleys.
375		*Melampyrum cristatum* L.	in the steppes, in birch-aspen and birch forests.

376	*Odontites vulgaris* Moench [*O. serotina* (Lam.) Dumort.]	in damp meadows, along roads, in damp places.
377	*Pedicularis achilleifolia* Steph.	rocky and gravelly slopes of hills.
378	*Pedicularis dasystachys* Schrenk	in a meadow in the vicinity of Mount Auliet.
379	*Pedicularis physocalix* Bunge	in meadows, near thickets of bushes.
380	*Rhinanthus minor* L.	through the meadows around the springs
381	*Scrophularia umbrosa* Dumort. [S. *alata* Gilib.]	in alder and willow groves along the banks of rivers.
382	*Verbascum phoeniceum* L.	in the steppes, in thein thickets of bushes, on meadow slopes
383	*Verbascum thapsus* L.	on rocky and gravelly slopes, in crevices of granite slabs in pine forests.
384	*Veronica anagallis-aquatica* L.	in river valleys and near springs, in water, in willows and aldersnikah
385	*Veronica dillenii* Crantz	on fine-grained areas among granite slabs in the western partpark style.
386	*Veronica incana* L.	in dry pine forests in crevices of granite slabs, in the steppes, in the bushold growth thickets.
387	*Veronica longifolia* L.	in alder forests, in willowsin wet pine forests, in bushesin the thickets.

388		*Veronica krylovii* Schischk.	in the meadows [Kara[Mysheva, Rachkovskaya, 1973].
389		*Veronica perpusilla* Boiss.	in alder forests, in thickets of bushes.
390		*Veronica pinnata* L.	on steppe and crushed soilslow slopes.
391		*Veronica scutellata* L.	in the clearings in the pine forests
392		*Veronica spicata* L.	in the pine forests in the creviceslines of granite slabs, in the steppes on rocky slopes.
393		*Veronica spuria* L.	in birch-aspen forests, on meadow steppes, in pine forests, ingrowths of steppe shrubs.
394		*Veronica verna* L.	edges of birch forestsowls, around the springs.
395	Orobanchaceae Vent.	*Orobanche caesia* Reichenb.	in steppes and meadows. Parasitizes on species of wormwood.
396	Plantaginaceae Juss	*Plantago major* L.	in alder forests along the berethe din of rivers, in damp forests, less often in the steppes.
397		*Plantago salsa* Pall. [P. *maritima* L.]	on soin the meadows, along the shores of lakes.
398		*Plantago maxima* Juss. ex Jacq.	in the alder forestin floodplain meadows, in bush thicketscove.
399		*Plantago media* L.	in the meadow steppes, in the aldersnikah, in damp pine forests, on the slopes
400		*Plantago urvillei* Opiz [P. *stepposa* Kupr.]	in the steppes, in alder forests, in pine forests, in busheskah.

401	Lamiaceae Lindl	*Dracocephalum nutans* L.	in the steppes, on the rubblelow slopes, in pine forests in cracks graniteslabs.
402		*Dracocephalum ruyschiana* L.	on steppein meadows, in steppes, in thickets of bushes, in pine forests.
403		*Dracocephalum thymiflorum* L.	in the steppes, on steppe meadows, in thickets of bushes, in pine forests.
404		*Galeopsis bifida* Boenn.	in alder forests, on forest edges, along roads, in littered areasfear.
405		*Glechoma hederacea* L.	in alder forests, along river banks, in damp meadows.
406		*Hyssopus ambiguus* (Trautv.) Iljin	on the rockon rocky and gravelly slopes in crevices of the granitethread slabs.
407		*Hyssopus macranthus* Boriss.	on the nakedgranite in the crevices.
408		*Leonurus glaucescens* Bunge	on slopes, in river floodplains, in thickets of steppe shrubs, in valleys between hills.
409		*Lophanthus schrenkii* Levin	inside the stonegrottoes, under the rocks.
410		*Lycopus europaeus* L.	in alder forests, in pine-birch forests, in willow groves.
411		*Lycopus exaltatus* L. fil.	in meadows, around springs, in stream floodplains.
412		*Mentha arvensis* L.	in alder forests, in dampin pine forests, in thickets of bushes.

413	*Nepeta pannonica* L.	on meadow and steppe mountain slopes, in thickets of bushes, on the edgeforests.
414	*Nepeta ucranica* L.	across the meadow steppes
415	*Phlomoides tuberosa* (L.) Moench *[Phlomis tuberosa* L.]	in steppe meadows, in steppes, in shrub thickets.
416	*Salvia deserta* Schang.	on the steppe-covered slopesgah, on mountain slopes, on forest edges, in the steppes.
417	*Salvia stepposa* Des.-Shost.	in the steppes, in Kuold growth thickets.
418	*Scutellaria galericulata* L.	in alder forests, in willow groves, in damp pine-birch forests, in thickets of bushes.
419	*Scutellaria supina* L.	rocky slopes of hills.
420	*Stachys palustris* L.	in damp meadows, in the forestin the pine-birch forests.
421	*Stachys sylvatica* L.	in the alder forests, in the willowska, along the banks of rivers, in pine forests on shady slopes.
422	*Thymus mongolicus* (Ronn.) Ronn. *[Th. asi- aticus* Serg.]	in cracks of granite slabs, under stones.
423	*Thymus marschallianusV/iWd.*	on steppein meadows, in steppes, in steppe shrub thickets.
424	*Thymus minussinensis* Serg.	in the crevices of granite slabs, on rocky and gravelly slopes along the shore of Lake Sabandykol.

425		*Ziziphora bungeana* Juz.	on rocky mountain slopes, in dry steppes.
426		*Ziziphora clinopodioides* Lam.	on graniteon rocky cliffs, in sparse forests and in rocky areassnapshots.
427	Campanulaceae Juss.	*Adenophora lilifolia* (L.) A.DC.	in birch and aspen forests, in shrub thickets.
428		*Campanula sibirica* L.	in the steppes, in thickets of bushes.
429	Asteraceae Dumort	*Achillea asiatica* Serg.	in the steppes and on the steppein the meadows [Karamysheva, Rachkovskaya, 1973].
430		*Achillea millefolium* L.	along the edges of birch forests.
431		*Achillea nobilis* L.	in the steppes, in the thickets of kuold trees, in pine forests on rocky soils, in weedy places along roads.
432		*Achillea salicifolia* Bess. *[Ptarmica salicifolia* (Bess.) Serg.]	meadows along the banks of springs
433		*Achillea setaceae* Waldst. et Kit.	on the rocklow slopes, along the edges of birch forests.
434		*Achorophorus maculatus* (L.) Scop.	in the damppine forests, along forest edges, in shrubberyin the thickets.
435		*Acroptilon repens* (L.) DC.	on fallow lands, near housing, roadsides, in fields and pasturesbischah.

436	*Ajania fruticulosa* (Ledeb.) Poljakov	on both sidesrank of the road in the surrounding area. Bayanaul village.
437	*Ancathia igniaria* (Spreng.) DC.	30 km southnot far from the village of Bayanaul, on the slopes of the hills near Lake Tuzkol.
438	*Antennaria dioica* (L.) Gaertn.	in dryin new forests, less often on rocky slopes of low mountains, in shrub steppes.
439	*Arctium leiospermum* Juz. et C. Sergievska	near housing, in waste places.
440	*Arctium tomentosum* Mill.	in alder forests, in damp pine forests, along roads, on heapsrented places.
441	*Artemisia abrotanum* L.[A. *procera* Willd., *A. proceraeformis* Krasch.]	on drying watersto the rivers, near the housing.
442	*Artemisia absinthium* L.	through the meadows, near the farmlya, in weedy places.
443	*Artemisia armeniaca* Lam.	in the steppes, on steppe meadows, in thickets of steppe bushesold trees, in pine forests on rocky slopesnah.
444	*Artemisia austriaca* Jacq.	in the steppes, on the salt flatson fertile soils near lakes and roads.
445	*Artemisia commutata* Bess.	in steppe pine forests, in the steppes.

446		in the pine forests, in thein the growths of steppe shrubs, in the steppes, in the coastin the forests, in littered places along the roads.
	Artemisia dracunculus L.	
447	*Artemisia frigida* Willd.	in caragana-wormwood steppes, in crevices of granite slabs, in pine forests on rocky slopes.
448	*Artemisia glabella* Kar. et Kir.	along the slopes of granite lowlands.
449	*Artemisia gmelinii* Web. ex Stechm. s.l.	on the edges of small-leaved forests.
450	*Artemisia laciniata* Willd.	on steppe meadows, on saline soils, in bush thicketsold people.
451	*Artemisia macrantha* Ledeb.	in the steppes, in meadows, in thickets of bushes, in crevices of granite rocks.
452	*Artemisia marschalliana* Spreng.	to the steppein sparse pine forests, on rocky and gravelly slopes.
453	*Artemisia nitrosa* Web. Ex Stechm.	Solontsy, banks of saline reservoirs, in the steppes on saline soils.
454	*Artemisia pontica* L.	along the edges of the small forestsforests.
455	*Artemisia radicans* Kupr.	along the banks of the stepperivers on soils of light mechanical soilstav.
456	*Artemisia rupestris* L.	in the meadows around the familyniks on slightly saline soil.

457	*Artemisia* sericeaWeberexStechm.	in the steppein dense pine forests and sparse woodlands, in mixed-grass feather-grass steppes, in thickets of steppe shrubsnikki
458	*Artemisia sieversianaWiWd.*	in steppe and saline meadows, in dry birch and pine forestsin the forests on rocky slopes.
459	*Artemisia schrenkiana* Ledeb.	by solontsam, sometimes around springs on saline soils.
460	*Artemisia scoparia* Waldst. et Kit.	near housing, in waste places.
461	*Artemisia sublessingiana* (B. Keller) Krasch. ex Poljak.	30 km south of the village of Bayanaul, on the slopethere are hills near Lake Tuzkol.
462	*Artemisia succulenta* Ledeb	found oneevery day N.N. Tsvelev in a solonetzic meadow [Karamysheva, Rachkovskaya, 1973].
463	*Artemisia tomentella* Trautv.	in the cracks of granite rocks.
464	*Artemisia vulgaris* L.	in alder forests, among shrub thickets, in damp pine forests.
465	*Aster altaicus* Willd. *[Heteropappus altaicus* (Willd) Novopokr.]	on rocky and gravelly slopes, on steppe meadows, in caragana-wormwood steppes.
466	*Aster biennis* Ledeb. *[Heteropappus biennis* (Ledeb.) Tamamsch.]	deposits, near roads.
467	*Aster tripolium* L. *[Tripolium vulgare* Nees.]	lake shores, on saline soils.
468	*Bidens radiata* Thuill.	near bodies of water, in small bays of lakes, around springs.

469	*Bidens tripartita* L.	in alder forests, in willow thickets along the banks of rivers.
470	*Brachyactis ciliata* (Ledeb.) Ledeb.	by fromshallows of lakes.
471	*Carduus crispus* L.	in aldersalong streams, in bush thickets, in damp pine forests, in weedy places, along roads.
472	*Carduus nutans* L.	along the roadsides, in steppe depressions, in rock crevices.
473	*Centaurea adpressa* Ledeb.	in the steppes, ason the hillsides, in shrubby thickets.
474	*Centaurea apiculata* Ledeb.	in the steppein the lowlands, at the foot of treeless hills.
475	*Centaurea scabiosa* L.	in forest clearings, in sparse pine forests, in fallow lands and along the roadsidesis dear to us.
476	*Centaurea sibirica* L.	on the slopes with stonesstony and gravelly soils, in feather grass bedspyakh, on granite slabs in pine forests.
477	*Chamomilla suaveolens* (Pursh) Rydb	near housing, in waste places.
478	*Chartolepis intermedia* Boiss.	on the salt lickcottony meadows.
479	*Chondrilla brevirostris* Fisch. et C. A. Mey.	along roadsides, dry stream beds.
480	*Chondrilla juncea* L.	roadsides.
481	*Cichorium intubus* L.	in meadows, near housing, along roadsides.
482	*Cirsium esculentum* (Sievers) C.A. Mey.	along the edges of small-leaved forests.

483	*Cirsium incanum* (S.G.Gmel.) Fisch.	in olto the hovelers along the banks of rivers, in damp pine forestsin open forests, in weedy places.
484	*Cirsium setosum* (Willd.) Bess.	floodplains ruwhose, edges of small-leaved forests, fallow lands, waste places.
485	*Cirsium vulgare* (Savi) Ten.	weedy places near housing.
486	*Crepis sibirica* L.	in the bush thicketsLyakh, in sparse forests.
487	*Crepis tectorum* L.	cracks in granite slabs, near housing, in weedy places.
488	*Crinitaria tatarica* (Less.) Czer. *[Linosiris ta- tarica* (Less.) C.A.Mey., *Galatella tatarica* (Less.) Novopokr.]	in the steppes.
489	*Crinitaria villosa* (L.) Grossh. *[Lynosiris villosa* (L.) DC.]	in the steppes, on rocky slopes.
490	*Echinops ritro* L.	in steppe meadows, in caragana-wormwood steppes, on gravelly slopes.
491	*Erigeron acer* L.	in pine forests, in thein the carnage, on the steppe slopes.
492	*Erygeron canadensis* L.	found near housing, in waste places, in place of old wintermokov.
493	*Erigeron lonchophyllus* Hook.	on the stoneon rocky and gravelly slopes, in steppe meadows.
494	*Erigeron podolicus* Bess.	meadows and glades

495	*Filago arvensis* L.	on fine-grained areas among sparse pine forest
496	*Galatella angustissima* (Tausch) Novopokr.	in the steppes, on rocky slopes, in thickets of bushes
497	*Galatella biflora* (L.) Nees	chalk along the edgesdeciduous forests.
498	*Galatella fastigiiformisNovopokr.*	by solontsevaty, natural meadows.
499	*Galatella hauptii* (Ledeb.) Lindl.	in resolutionin the burned pine forests.
500	*Galatella punctata* (Waldst. et Kit.) Nees	in alder and willow groves along the banks of streams and genusniks.
501	*Gnaphalium kasachstanicum* Kirp.	by notelyam and clay banks of reservoirs.
502	*Helichrysum arenarium* (L.) Moench	at oncethin pine forests, on rocky slopes.
503	*Hieracium porphyrii* Schischk et Serg.	on the edges of small-leaved forests.
504	*Hieracium robustum* Fr.	in sparse spacespictures on granites.
505	*Hieracium umbellatum* L.	in the cracks of the stonefilament plates, on steppe meadows, in sparsepine forests.
506	*Hieracium virosum* Pall.	in the steppes, thickets of bushes, in pine and birch forests.
507	*Inula aspera* Poir.	on steppe slopes, in forb-wormwood steppes, on solonetz soilsin these meadows.
508	*Inula britannica* L.	in the steppes, along the shores of lakes on saline soils.

509	*Inula caspica* Blume	along road ditches, in natural meadows.
510	*Inula hirta* L.	in pine forests on morefertile soils.
511	*Inula salicina* L.	small-leaved along the edgesforests.
512	*Jurinea multiflora* (L.) B.Fedtsch.	in the steppes, on steppe meadows, on rocky slopes.
513	*Lactuca serriola* Torner ex L.	near housing, on fallow lands, on roadsides, in weedy placesstam.
514	*Lactuca tatarica* (L.) C.A. Mey.	along the shores of lakes, in meadows with saline soils, nearhorn.
515	*Ligularia macrophylla* (Ledeb.)DC.	around springs, in meadows around shrubberygrow.
516	*Ligularia sibirica* (L.) Cass.	in alder forests along the banks of streams and springs, in damp meadows.
517	*Ligularia thyrsoidea* (Ledeb.) DC.	along the edges of small-leaved forests.
518	*Matricaria inodora* L. *[Tripleurospermum in- odorum* (L.) Sch. Bip.]	near housing, in fallow lands, on the edges of deciduous forests, in waste places.
519	*Phalacrachena calva* (Ledeb.) Iljin	on thespring meadows, on the slopes of hills.
520	*Picris hieracioides* L.	through meadows, along roadsides.
521	*Pilosella asiatica* (Naeg. et Peter) Schljak. *[Pi- losella echioides* (Lumn.) F.Schultz et Sch.Bip.]	through the meadows, in the pine forests.

522	*Saussurea amara* (L.) DC.	on saline and saline meadows.
523	*Saussurea salsa* (Pall.)Spreng.	on solonetz and saline soils.
524	*Scorzonera austriaca* Willd.	in the steppes on kamelancholy slopes.
525	*Scorzonera crispa* M.Bieb.	on the slopes under granite outcrops in the vicinity of Lake Zhasybay
526	*Scorzonera ensifolia* M.Bieb.	in the meadows and steppes.
527	*Scorzonera parviflora* Jacq.	along the edges of small-leaved forests.
528	*Scorzonera purpurea* L.	in steppe meadows, in mixed-grass-feather-grass steppes, inin new forests, on grassy slopes.
529	*Scorzonera stricta* Hornem.	on the slopes, under granite outcrops in the vicinity of Lake Zhasybye.
530	*Senecio dubius* Ledeb.	on salt licks, near housing, in waste places.
531	*Senecio erucifolius* L.	in the alder forestscircle of streams and springs, in damp pine forests, in shrub thickets, in birch-aspen forests.
532	*Senecio jacobaea* L.	in the steppes, on the steppein the meadows, on the steppe slopes.
533	*Senecio vulgaris* L.	in sparse pine forestsin the forests.

534	*Serratula cardunculus* (Pallas) Schischk.	on the slopes, under granite outcrops in the vicinity of Lake Zhasybay, on rocky steppe slopes in the vicinity of Mount Auliet.
535	*Serratula coronata* L.	in alder forests, in damp pine forests, on steppe meadowsgam and mountain slopes.
536	*Solidago dahurica* Kitag. [S. *virgaurea* L.]	in humid pine forests, in mesophilic forestsgrowths of bushes.
537	*Sonchus arvensis* L.	shores of lakes, rivers, in damp meadows, near dwellings, in fallow lands, in weedy places.
538	*Sonchus oleraceus* L.	in the alder forests, in thelittered areas, near housing and roads.
539	*Sonchus palustris* L.	northern slope, aboutgrown to the lake Turaigyr, in the black alder forest [Karamysheva, Rachkovskaya, 1973].
540	*Tanacetum santolina* C. Winkl.	across the steppes on slightly saline soil.
541	*Tanacetum vulgare* L.	in the steppes, on steppe slopes, in littered areas near roads.
542	*Taraxacum erytrospermum* Andrz.	according toin lush meadows, inter-hill hollows, on the slopes of hills and low mountains.
543	*Taraxacum glaucanthum* (Ledeb.) DC.	on salt licks and salt marshes.
544	*Taraxacum leucanthum* (Ledeb.) Ledeb.	on salt licks and salt marshes.

545			*Taraxacum officinale* F.H. Wigg.	along roads, in forests, in meadows.
546			*Tragopogon capitatus* S.A. Nikitin	in pineon the rocky slopes of the low mountains, in theold growth thickets.
547			*Tragopogon orientalis* L.	in meadows and forest edgeskah.
548			*Youngia diversifolia* (Ledeb.) Ledeb. *[Crepis diversifolia* (Ledeb.) B. Fedtsch.]	in the cracks of granite rocks.
549			*Xanthium strumarium* L.	damp, salted meadows, roadsides, weedy places.
550	LILIOPSIDA	Butomaceae Rich	*Butomus umbellatus* L.	on the diseased moongah, by the water.
551		Alismataceae Vent.	*Alisma gramineum* Lej.	along the shores of lakes, in meadows.
552			*Alisma plantago-aquatica* L.	on wet moonsgah, in the coastal water strip of lakes.
553		Juncaginaceae Rich.	*Triglochin maritimum* L.	on salt licks and salt lickschakas along the banks of rivers, lakes, and in damp meadows.
554			*Triglochin palustre* L.	along the shore of the lake SabanDykol, near the village of Bayanaul.
555		Potamogetonaceae Dumort.	*Potamogeton pectinatus* L.	lake Sabandykol, near the village. Bayanaul, in the water.
556		Iridaceae Juss.	*Iris halophila* Pall.	in saline meadows, near lakes, in saline areas, in thickets of bushes.
557			*Iris lactea* Pall.	in the meadows

558		*Iris scariosa* Willd. ex Link	in the rocky steppes
559	Liliaceae Juss.	*Fritillaria meleagroides* Patrin ex Schult. & Schult, f.	in a meadow in the vicinity of Mount Auliet.
560		*Gagea emarginata* Kar. et Kir	through deep ravines, in thickets of bushes.
561		*Gagea granulosa* Turcz.	in a meadow in the vicinity of Mount Auliet, in alder forests in the western part of the park.
562		*Gagea fedtschenkoana* Pascher	in thickets of bushes, on steppe slopes.
563		*Gagea filiformis* (Ledeb.) Kun th.	on the rockmuddy and gravelly soils, in bush thicketsnikov [Karamysheva, Rachkovskaya, 1973].
564		*Tulipa patens* C. Agardh ex Schult. & Schult. f.	in dry feather grass steppes, on steppe meadows, on gravelly slopes
565	Alliaceae J. Agardh	*Allium coeruleum* Pall.	thickets of bushes on the slopes of the hills.
566		*Allium globosum* M.Bieb. ex Redoute	on kaon variegated soils, in pine forests on slopes, less often in thickets of shrubs
567		*Allium flavescens* Besser	in feather grasspah, on rocky slopes.
568		*Allium hymenorrhizum* Ledeb.	on the naturewhose and spring meadows, near the bushcove.
569		*Allium lineare* L.	in pine forests on rocky slopes.
570		*Allium nutans* L.	in the steppes, in the pine forests on the bordernitah.
571		*Allium obliquum* L.	through meadows, forest edgesstream thickets of bushes.

572			*Allium oliganthum* Kar. et Kir.	by timewatercourses on granites.
573			*Allium pallasii* Murr.	on rocky slopes in the environs. lake Toraigyr.
574			*Allium rubens* Schrad. ex Willd.	in mixed-grass steppes, in pine forests on the mountainson the slopes and trails of low graniteryi.
575			*Allium strictum* Schrad.	on steppe meadows, rocky mountain slopes, in bushesold trees and pine forests.
576			*Allium tulipaefolium* Ledeb.	thickets of kuold trees, saline soils.
577		Asparagaceae Juss.	*Asparagus brachyphyllus* Turcz.	salt licks, salt marshes.
578			*Asparagus neglectus* Kar. et Kir.	on the stonesteep slopes in the surroundings lake Zhasybay.
579			*Asparagus officinalis* L.	in Zaroeli Kustarniks, on steppe meadows, in birch groveska, sometimes in pine forests.
580		Orchidaceae Juss.	*Dactylorhiza incarnata* (L.) Soa	in raw axlesnew and birch groves.
581			*Dactylorhiza fuchsii* (Druce) Soo	banks of the ruwhose, wet meadows on the outskirts of swamps andspring meadows.
582			*Malaxis monophyllos* (L.) Sw.	along the valleys of streams in alder and birch forests.
583			*Neottianthe cucullata* (L.) Schltr.	in shady pine forests.

584		Juncaceae Juss.	*Juncus atratus* Krock.	in meadows, in willow thickets [Karamysheva, Rachkovskaya, 1973].
585			*Juncus bufonius* L.	in damp places, along old abandoned roads.
586			*Juncus gerardii* Loisel.	edges of salt marshes and salt marshes, shores of lakes and rivers, wet meadows.
587			*Juncus nastanthus* V. K.recz. et Gontsch.	no shore of the lake Sabandykol, near the village. Bayanaul.
588			*Juncus ranarius* Song, et Perrier ex Bilott.	in meadows, in damp places.
589			*Juncus tenuis* Willd.	along the shore of the lake Zhasybye.
590			*Luzula pallescens* Sw.	in damp meadows, in willow groves, in alder forests, along the banks of rivers.
591		Cyperaceae Juss	*Bolboshoenus maritimus* (L.) Palla	along the shoregam of natural and artificial reservoirs, in swamps, in wet meadows on saline soils.
592			*Carex acuta* L.	in alder forests, on damp meadowsgah.
593			*Carex acutiformis* Ehrh.	in alder forests, in damp meadows, in willow thickets.
594			*Carex caryophyllea* Latourr.	on slopes, in thickets of bushes and in pine forests withcom.
595			*Carex diandra* Schrenk	in the swampy forestssakh [Karamysheva, Rachkovskaya, 1973].

596		*Carex diluta* M.Bieb.	in alder forests along mountain rivers, in damp meadows.
597		*Carex disticha* Huds.	in the meadows in the western part of the park.
598		*Carex elongata L.*	raw black alder (according to collections by N.N. Tsvelev) [Karamysheva, Rach[Kovskaya, 1973].
599		*Carex macroura* Meinsh.	Pine forest on the slope at the foot of Mount Auliet.
600		*Carex pallescens* L.	slope to Lake Zhasybay with granite outcrops.
601		*Carex pediformis* C.A. Mey.	in the richly varied grass-feather grass steppes, in the shrubthickets, sometimes in pine forests.
602		*Carex praecox* Schreb.	Pine forest on the slope at the foot of Mount Auliet.
603		*Carex pseudocyperus* L.	in swampy aspen groves, in alder forests along the banks of mountain rivers.
604		*Carex riparia* Curtis	in alder forests along the berethe din of mountain rivers, in the steppe depressions.
605		*Carex secalina* Willd. ex Wahl enb.	along the shore of the lake Toraigyr.
606		*Carex songorica* K.ar. et K.ir.	in the swampin aspen groves, in meadows, in alder forests along the banks of rivers.
607		*Carex stenophylla* Vachleb.	on a rocky slope in the vicinity. lake Toraigyr.

608		*Carex supina* Willd. ex Wahlenb.	in pinein bushes, in shrub thickets, in mixed-grass feather-grass steppes.
609		*Carex vesicaria* L.	along the shore of the lake Toraigyr, on a damp meadow in the vicinity. Auliet Mountains.
610		*Eleocharis palustris* (L.) Roem et Schult.	on the banks of rivers and lakes, in wet natural areasin the meadows.
611		*Eleocharis uniglumis* (Link) Schult.	in Olkhovin the nikahs and willow groves along the banks of rivers.
612		*Scirpus sylvaticus* L.	in alder forests along the berethe din of streams in damp forests.
613		*Scirpus tabernaemontani*C.C.Gmel.	in the water, along the banks of lakes and streams.
614	Poaceae Barnhart	*Achnatherum splendens* (Trin.) Nevski [Lasia-*grostis splendens* (Trin.) Kunnth.]	in dry wallspah
615		*Agropyron cristatum* (L.) Beauv.	slope to Lake Zhasybay with granite outcrops.
616		*Agropyron pectinatum* (Bieb.) Beauv.	aboutroad ranks, deposits.
617		*Agrostis gigantea* Roth	in meadows, in thickets of bushes on the outskirts of springs and mountain streams.
618		*Agrostis stolonifera* L.	in damp meadows, in the valleys of mountain streams, along forest edges.

619	*Agrostis vinealis* Schreb. *[Agrostis syreistsch-ikowiiP.* Smirn]	in the meadows around the springs (Bayanaul Mountains) [Karamysheva, Rachkovskaya, 1073].
620	*Agrostis tenuis* Sibth.	in damp meadows, on the edges of forest stands.
621	*Alopecurus aequalis* Sobol.	along the banks of rivers, lakes, in bushes, along thein some places.
622	*Alopecurus arundinaceus* Poir.	along the shorethe din of streams, damp meadows, and mixed-grass steppes.
623	*Alopecurus pratensis* L.	on the wet moonsgah, along the banks of streams and springs, along the westdynamo.
624	*Avena fatua* L.	roadsides, fallow lands, waste places.
625	*Beckmannia syzigachne* (Steud.) Fernald	through damp meadows, along the banks of streams in the blackalder forests.
626	*Brachypodium pinnatum* (L.) Beauv.	on the moonin the clearings, in the thickets of bushes, in thein the forests, on the edges of forests.
627	*Bromopsis inermis* (Leyss.) Holub	on the steppemeadows, in thickets of bushes, in drysnapshots.
628	*Calamagrostis epigeios* (L.) Roth	in the meadowgrass, mixed-grass-feather grass and fescue steppes.

629	*Calamagrostis langsdorffii* (Link) Trin. [C. *purpurea* (Trin.) Trin. subsp. *langsdorffii* (Link.) Tz- vel.]	in alder forests, in bush thickets, in birch-pine forests.
630	*Cleistogene kitagavae* Ohwi	between graniteslabs
631	*Dactylis glomerata* L.	natural meadows.
632	*Echinochloa crusgali* (L.) Beauv.	near the houselya, in weedy places.
633	*Elymus caninus* (L.) L. []	in the alder forests, in thein thickets of bushes, in steppe clearings.
634	*Elymus viridiglumis* (Nevski) Czerep.	in betweenmountain basins, in thickets of bushes.
635	*Elytrigia lolioides* (Kar. et Kir.) Nevski	kuold growth thickets, rocky slopes, behindlie down.
636	*Elytrigia repens* (L.) Nevski	in alder forests, in meadows, in steppes.
637	*Eragrostis pilosa* (L.) Beauv.	in the cracks of granite slabs and in weedy places.
638	*Festuca rubra* L.	on natural meadowsgah.
639	*Festuca rupicola* Heuff.	on rocky slopes in the environs. lake Zhasybay.
640	*Festuca valesiaca* Gaudin	in the steppes, on dry and saline meadows, in dry pine forests and sparsewheels.
641	*Helictotrichon desertorum* (Less.) Nevski	in dry steppes and on mountain slopes.
642	*Helictotrichon schellianum* (Hack.) Kitag.	in steppe meadows and steppes.
643	*Hierochloe odorata* (L.) P.Beauv.	on the steppemeadows.
644	*Hordeum bogdanii* Wilensky	saline meadows.

645	*Hordeum brevisubulatum* (Trin.) Link	by berivers, lakes, and saline meadows.
646	*Hordeum jubatum* L.	roadsides, near housing, fallow lands, in waste places.
647	*Koeleria cristata* (L.) Pers.	on rocky slopes in the environs. Lake Zhasybay.
648	*Leymus angustus* (Trin.) Pilg.	on solonetz, saline lake shores, steppe terracesrivers, in steppe depressions.
649	*Leymus paboanus* (Claus) Pilg.	in the steppe, on saline soils, solonetz.
650	*Leymus ramosus* (Trin.) Nevski	in the steppes, on salted meadows, in depressions, on roadsides.
651	*Melica altissima* L.	in the bushes of the bushkov, along deep ravines, along pine sparse forestssyam.
652	*Melica nutans* L.	birch and aspen forests along streams.
653	*Melica transilvanica* Schur.	shore of lake ZhasyBai, sanatorium "Beryozka".
654	*Panicum miliacum* subsp. *ruderale* Kita	along roads, near housing, along the shores of lakes, in wastelands.
655	*Phalaroides arundinacea* (L.) Rauschert	along the banks of streams and in damp meadows.
656	*Phleum phleoides* (L.) H.Karst.	in meadow steppes, on rocky mountain slopes.
657	*Phragmites australis* (Cav.) Trin. ex Steud.	along the shores of lakes, in black alder forests, in thicketsa field of bushes.

658	*Poa angustifolia* L.	in the meadows and steppes, in thegrowths of mesophilic shrubs, in black woodkhovniki.
659	*Poa annua L.*	in the meadows, by the roads.
660	*Poa bulbosa* L.	on rocky slopes in the environs. lake Zhasybay.
661	*Poa nemoralis* L.	in the forests on the low slopesmountains and bushes.
662	*Poa palustris* L.	in damp willows and birchesnyakah.
663	*Poa pratensis* L.	across the meadows, along the mountain banksrivers.
664	*Poa stepposa* (Kryl) Roshev.	on stonein dry steppes, in dry pine forests, on dry steppe slopes.
665	*Poa urssulensis* Trin.	in alders, in kuold trees, forest clearings, rocky slopes and pebbles.
666	*Psathyrostachis juncea* (Fisch.) Nevski	withmeadows, fallow lands, roadsides.
667	*Puccinellia distans* (Jacq.) Pari.	Solonetsevagreen meadows, near the houses.
668	*Puccinelia giganthea* (Grossh.) Grossh.	as part of saline meadows, along the banks of rivers and lakes.
669	*Puccinelia hauptiana* V. Crecz.	on the outskirtssalt licks for us.
670	*Puccinellia tenuissima* Litv. ex K.recz.	on soin the meadows near the lakes, in the thickets of iris
671	*Puccinellia tenuiflora* (Griseb.) Scribn. et Merr.	in saline meadows near lakes, in thicketsiris's lyakh.

672		*Scolochloa festucaceae* (Willd.) Link.	takehectares of lakes, floodplains, swamps.
673		*Setaria viridis* (L.) P.Beauv.	in the steppesocieties, in cracks in granite slabs, in weedy places.
674		*Stipa capillata* L.	in mixed-grass steppes, on rocky slopes.
675		*Stipa pennata* L.	in dry steppes, less often in pine forests.
676		*Stipa tirsa Steven* [S. *stenophylla* (Lindem.) Trautv.]	in the steppes, along the ravines.
677		*Stipa zalesskii* Wilensky	along the slopespok.
678	*Typhaceae* Juss.	*Typha angustifolia* L.	along the banks of rivers and lakes.
679		*Thypha latifolia* L.	the banks of the springs, behindpond, reservoir.
